# Optimizing purebred selection to improve crossbred performance

**DOI:** 10.3389/fgene.2024.1384973

**Published:** 2024-09-24

**Authors:** Somayeh Barani, Sayed Reza Miraie Ashtiani, Ardeshir Nejati Javaremi, Majid Khansefid, Hadi Esfandyari

**Affiliations:** ^1^ Department of Animal Science, Faculty of Agriculture, College of Agriculture and Natural Resources, University of Tehran, Karaj, Iran; ^2^ Agriculture Victoria Research, AgriBio, Centre for AgriBioscience, Bundoora, VIC, Australia; ^3^ School of Applied Systems Biology, La Trobe University, Bundoora, VIC, Australia; ^4^ Qualitas, Zug, Switzerland

**Keywords:** crossbred performance, genetic correlation between crossbred and purebred populations, unknown parent group, metafounders. ssGBLUP, SSGblup

## Abstract

Crossbreeding is a widely adopted practice in the livestock industry, leveraging the advantages of heterosis and breed complementarity. The prediction of Crossbred Performance (CP) often relies on Purebred Performance (PB) due to limited crossbred data availability. However, the effective selection of purebred parents for enhancing CP depends on non-additive genetic effects and environmental factors. These factors are encapsulated in the genetic correlation between crossbred and purebred populations ( $r_{pc}$ ). In this study, a two-way crossbreeding simulation was employed to investigate various strategies for integrating data from purebred and crossbred populations. The goal was to identify optimal models that maximize CP across different levels of $r_{pc}$ . Different scenarios involving the selection of genotyped individuals from purebred and crossbred populations were explored using ssGBLUP (single-step Genomic Best Linear Unbiased Prediction) and ssGBLUP-MF (ssGBLUP with metafounders) models. The findings revealed an increase in prediction accuracy across all scenarios as $r_{pc}$ values increased. Notably, in the scenario incorporating genotypes from both purebred parent breeds and their crossbreds, both ssGBLUP and ssGBLUP-MF models exhibited nearly identical predictive accuracy. This scenario achieved maximum accuracy when $r_{pc}$ was less than 0.5. However, at $r_{pc}$ = 0.8, ssGBLUP, which exclusively included sire breed genotypes in the training set, achieved the highest overall prediction accuracy at 73.2%. In comparison, the BLUP-UPG (BLUP with unknown parent group) model demonstrated lower accuracy than ssGBLUP and ssGBLUP-MF across all $r_{pc}$ levels. Although ssGBLUP and ssGBLUP-MF did not demonstrate a definitive trend in their respective scenarios, the prediction ability for CP increased when incorporating both crossbred and purebred population genotypes at lower levels of ${\;r}_{pc}$ . Furthermore, when $r_{pc}$ was high, utilizing paternal genotype for CP predictions emerged as the most effective strategy. Predicted dispersion remained relatively similar in all scenarios, indicating a slight underestimation of breeding values. Overall, the $r_{pc}$ value emerged as a critical factor in predicting CP based on purebred data. However, the optimal model to maximize CP depends on the factors influencing $r_{pc}$ . Consequently, ongoing research aims to develop models that optimize purebred selection, further enhancing CP.

## 1 Introduction

Crossbreeding in livestock and poultry breeding is used to maximize crossbred performance (CP) through benefiting from heterosis and breed complementarity. Genetic evaluation in the crossbreeding system is usually based on phenotypes and genotypes of purebred parents, which in fact leads to the selection of the best purebred parents in the pure populations. However, the best pure parents will not necessarily produce the optimal crossbred progenies, which means the performance of purebred parents is not the best predictor for CP (Esfandyari et al., 2015). Ideally, crossbred data should be used rather than purebred data which can lead to a more significant improvement in CP; nevertheless, gathering data from commercial populations can be challenging and expensive (Wei and van der Werf, 1994). Therefore, animal breeders inevitably utilize the performance of purebred parents to predict the CP.

The genetic correlation ( $r_{pc}$ ) between purebred and crossbred populations is a pivotal factor influencing the accuracy of predicting CP based on purebred data (Legarra et al., 2009). The value of $r_{pc}$ is shaped by various factors, including, (i) Genotype by genotype interaction (G × G); Arising from differing allelic frequencies of causal variants in purebred and crossbred populations (Christensen and Lund, 2010). (ii) Genotype by environment interaction (G × E); Resulting from environmental disparities between the two populations. (iii) Incompatibility of measured traits; Present in purebred and crossbred populations (Christensen, 2012). These factors, coupled with non-additive genetic effects, particularly dominance effects, can lead to deviations in $r_{pc}$ from 1. When $r_{pc}$ deviates from 1, utilizing purebred performance (PB) to assess crossbreds may hinder the rate of improvement in CP. Research suggests that if this deviation is attributable to dominance effects, implementing a dominance model can enhance the prediction accuracy of CP when the reference comprises purebred populations (Legarra et al., 2015). However, in practice, deviations in $r_{pc}$ from 1 stem from multiple factors mentioned earlier.

Another challenge in predicting CP is the low genetic relatedness between parental breeds, despite their shared common ancestors several generations ago. Addressing these complexities is crucial for refining predictive models and advancing the accuracy of CP evaluations based on purebred data.

In recent years, single-step genomic BLUP (ssGBLUP) has significantly increased the accuracy of breeding value estimation by blending marker and pedigree information of both genotyped and ungenotyped animals (Legarra et al., 2009; Christensen and Lund, 2010). However, the main remaining challenge is how to combine pedigree-based and genomic relationships, given the base population does not have pedigree or genotype information. In the same vein, Christensen constructed the marker-based relationship matrix by assuming that all allele frequencies equal to 0.5 and base animals are related and inbred (Christensen, 2012). Subsequently, Legarra et al. (2015) proposed a comprehensive theoretical framework for incorporating relatedness within and across founders into a base relationship matrix, using the concept of metafounders (MFs). The base animals assumed to be related, so in ssGBLUP with metafounders (ssGBLUP-MF), matrix G is constructed with all allele frequencies equal to 0.5. Consequently, the founders of the breeds are related to a common ancestor, represented by the matrix Γ which describes the relationship of the founders (Legarra et al., 2015).

Motivated by the challenges inherent in predicting CP, this paper strives to pinpoint the optimal strategy for integrating data and selecting the most suitable model to maximize CP at various levels of $r_{pc}$ . This objective remains irrespective of the factors causing deviations in $r_{pc}$ from 1.

## 2 Materials and methods

### 2.1 Simulation

**FIGURE 1:**
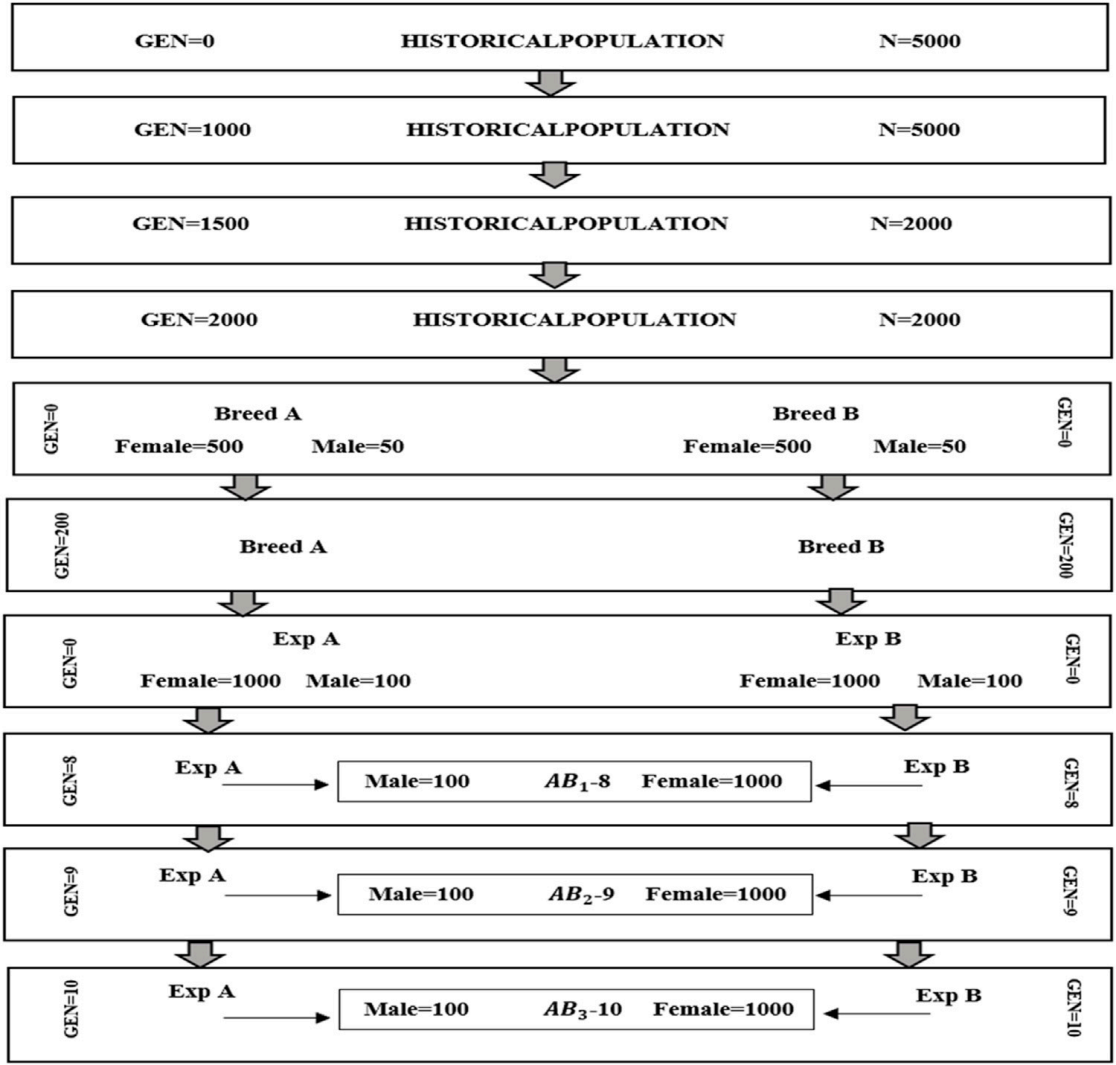
Schematic representation of the simulated population. A (paternal line) and B (maternal line) are purebred populations. ${AB}_{1}$ , ${AB}_{2}$ , and ${AB}_{3}$ are crossbred populations that are derived from generations (GEN) 8, 9 and 10 of expanded (EXP) purebred populations A and B.

The simulation was conducted in two parts. The first part was focused on generating datasets of purebred and crossbred animals for a single trait with a heritability of 0.3 using the QMSim software (Sargolzaei and Schenkel, 2009). In the initial phase of the simulation, a historical population of 5,000 founders was simulated for 1,000 generations with a constant population size of 5,000. This step aimed to establish genetic drift and linkage disequilibrium (LD) between markers and causal variants. Subsequently, the population size was gradually reduced from 5,000 to 2000 individuals over an additional 1,500 generations. After that, the population size was considered steady during the last 500 generations. To create two breeds (breeds A and B), two random samples of 50 males and 500 females were taken from the last generation of the historical population (the first bottleneck), then the population size was expanded over the next 200 generations. Simulating various population sizes and dynamics across generations aids in comprehending the impact of genetic drift, selection, and other evolutionary forces. The initial step involved simulating linkage disequilibrium between markers and causal variants. The subsequent step consisted of simulating a gradual reduction in population size resulting from domestication and other minor bottleneck events (Brito et al., 2011). The final step entailed creating two separate breeds. In this step, to simulate the sheep breeding system, we implemented random mating with 5 descendants per dam considering 1/2 of the twining average and 4 parturitions per generation. In the next step, the second bottleneck was simulated by selecting two random samples (100 males and 1,000 females) from the last generation of the breeds A and B to generate the recent populations of the breeds (called expanding A and B). In this step, breeding values were estimated based on best liner unbiased prediction (BLUP) method. The next generations were simulated by imposing a replacement rate of 20% for dams and 50% for sires. The culling and selection designs were contingent on age and high estimated breeding value, respectively. The breeding scheme in this step was continued for 10 generations. Then crossbred animals (F1) were produced from crossing between males from expanding A and females from expanding B populations. We simulated three crossbred populations (F1) that were generated from generations 8, 9, and 10 (Figure 1).

Genome structure was simulated with 26 autosomal chromosomes and a total length of 3,421 centimorgan (cM) similar to the sheep genome size estimated by Prieur et al. (2017). We simulated 50K single nucleotide polymorphisms (SNP) and 500 quantitative trait loci (QTLs) randomly scattered across the whole genome. Minor allele frequencies (MAF) of markers were assumed to be upper than 0.05 and mutation rates of the SNPs and QTL was $2.5\times 10^{-5}$ . The QTL allele effects were inferred from a Gamma distribution with α=0.4 (summarized in Table 1). True breeding values (TBVs) were obtained from the sum of the additive effect of QTL. The phenotypes were calculated based on the additive genetic and residual effects. Subsequently, we measured the fixation index ( $F_{st}$ ) and performed the principal component analysis (PCA) to assess the population structure and distinctness of the three populations. The $F_{st}$ is a measure of population differentiation due to genetic structure (Nagylaki, 1998). PCA is a multivariate analysis that reduces the dimensionality of the data while preserving their covariance which we applied to find the eigenvalues and eigenvectors of the covariance matrix of allele frequencies (Reich et al., 2008).

**TABLE 1:** The parameters used for simulating trait and genome.

Item	Value
Heritability (h^2^)	0.3
Genome size	3,421 cM
Number of chromosomes	26
Number of markers	50,000
Number of QTL	500
Mutation rate in QTL and markers	2.5 × 10^−5^
Minor allele frequency in QTL and markers	0.05
Distribution of additive QTL effects	Gamma (α = 0.4, 1.66)
Additive genetic correlation between purebred and crossbred populations ( $r_{pc}$ )	0.2, 0.5 or 0.8

In the second part of the simulation, to investigate the impact of $r_{pc}$ on the identification of optimal and cost-efficient strategies for improving CP, three levels of $r_{pc}$ (low, medium, and high, corresponding to 0.2, 0.5, and 0.8, respectively) were simulated using the BGLR and MASS packages in R (Ripley et al., 2013; Pérez and de Los Campos, 2014). To simulate the ${\;r}_{pc}$ levels, the additive effects of purebreds and crossbreds were simulated using a multivariate normal distribution with mean 0 and a positive-definite symmetric matrix specifying the covariance matrix of the variables, according to the desired levels of $r_{pc}$ and the effects of QTLs. Therefore the ${\;r}_{pc}$ is defined as follows (Falconer, 1996):

$$r_{pc}=\frac{\sigma_{A_{PB,CB}}}{\sigma_{A_{PB}}\sigma_{A_{CB}}}$$

where $\sigma_{A_{PB,CB}}$ is the additive genetic covariance between PB and CB performance, and $\sigma_{A_{PB}}$ and $\sigma_{A_{CB}}$ are the additive genetic standard deviation in purebred and crossbred populations, respectively.

We conducted the population simulation 10 times and subsequently performed a comparative analysis of the predictive abilities of three methods: BLUP-UPG (BLUP with unknown parent group), ssGBLUP, and ssGBLUP-MF methods. The breeding values were estimated based on the same pedigree and data file, considering four scenarios of genotype selection from purebred and crossbred populations: SC1: breeds A, B and crossbreds, SC2: breeds A and B, SC3: breed A and SC4: breed B population.

### 2.2 Data analysis

Three prediction models were used to calculate breeding values: BLUP-UPG, ssGBLUP, and ssGBLUP-MF. The generation 0 produced after the second bottleneck was assumed to be the base population (i.e., only the pedigree information after generation 0 was used to predict breeding values). For the ssGBLUP and ssGBLUP-MF models, we explored various scenarios for including genotypes from both purebred and crossbred populations. The statistical model was as follows:

y=Xb+Zu+e

where y is the vector of phenotypes, b is the vector of fixed effects (breed, sex, and generation) and u is the vector of additive genetic effects distributed as $u∼N\left(0,\sigma_{u}^{2}\right),$ X and Z are incidence matrices relating the animals to the fixed effects and the additive effects, respectively, e is a vector of residuals that were sampled from a normal distribution, with $e∼N\left(0,\sigma_{e}^{2}\right).$ We used the BLUPF90 family (Misztal et al., 2014) of programs, which includes airemlf90 and blupf90test, to estimate variance components using restricted maximum likelihood (REML). Moreover, we used predictf90 to adjust the phenotypes.

#### 2.2.1 Best Linear Unbiased Prediction (BLUP)

In this study, we utilized various advanced statistical models for genetic prediction. Each model has its own hypotheses, advantages, and limitations. One of the models we employed is BLUP-UPG, which incorporates unknown-parent groups (UPGs) to handle missing pedigree data. Accurate group assignments and variance estimation are crucial for this model to ensure robust predictions (Henderson, 1975). In our study, we encountered unknown parents only in the first generation, while we knew the parents in all subsequent generations. The use of UPGs in this model helps estimate the genetic merit of animals in the pedigree when their parents are unknown (Graser et al., 1987). Without defining UPGs for each breed, the genetic merit of animals with unknown parents is assumed to be equal to that of the base generation, potentially leading to biased predictions.

#### 2.2.2 Single-Step Genomic Best Linear Unbiased Prediction (ssGBLUP)

Single-step GBLUP model combines phenotypes, pedigree, and genotypes into a single evaluation and replaces the pedigree relationships between genotyped individuals with realized relationships by incorporating pedigree and genomic relationships in H matrix, but requires significant computational resources and high-quality data. The additive (***u***) and residual (***e***) effects are assumed to be independently distributed with $u∼N\left(0,{H\sigma }_{u}^{2}\right)$ and $e∼N\left(0,{I\sigma }_{e}^{2}\right)$ , respectively. The H matrix is described as:

$$H=\left[\begin{array}{cc} H_{11} & H_{12}\\ H_{21} & H_{22} \end{array}\right]=\left[\begin{array}{cc} A_{11}+A_{22}A_{22}^{-1}\;\left({G-G}_{22}\right)A_{22}^{-1}A_{21} & G_{12}A_{22}^{-1}G\\ GA_{22}^{-1}G_{11} & G \end{array}\right]$$

$$H^{-1}=A^{-1}+\left[\begin{array}{cc} 0 & 0\\ 0 & G^{-1}-A_{22}^{-1} \end{array}\right]$$

where the subscript 1 and 2 indicate ungenotyped and genotyped individuals, respectively. The H matrix can be constructed from the relationships of genotyped and ungenotyped animals (Aguilar et al., 2010). However, ***H*** ^*−**1***^ can be directly calculated using $A^{-1}$ and $G^{-1}$ which contain pedigree and genomic relationships related to ungenotyped and genotyped individuals, respectively, and $A_{22}$ is the pedigree relationship matrix just for genotyped animals.

#### 2.2.3 Single-Step Genomic BLUP with Metafounders (ssGBLUP-MF-)

A metafounder is a pseudo-individual considered both sire and dam of all base animals. This approach extends VanRaden`s method (VanRaden, 1992) for estimating relationships across breeds (Legarra et al., 2015). To further enhance prediction accuracy in diverse populations, we incorporated metafounders in the ssGBLUP-MF model. However, this increased the complexity of the model and posed challenges in parameter estimation (Legarra et al., 2015). The significance of the metafounder is particularly important when considering both racial groups together. The use of different scenarios also arises from the limitation of measuring certain traits in crossbred groups, so it is inevitably based on the parental group. In our simulation, the founders in the base population of purebred parents were defined based on the number of pure breeds, so we had two metafounders.

In ssGBLUP, $H^{-1}$ with metafounders $\left(H{\left(\Gamma \right)}^{-1}\right)$ can be represented as (Christensen et al., 2015):

$$H_{\left(\Gamma \right)}^{-1}=A_{\left(\Gamma \right)}^{-1}+\left[\begin{array}{cc} 0 & 0\\ 0 & {\tau \left({0.95G+0.05A}_{\left(\Gamma \right)22}\right)}^{-1}-{\omega A}_{\left(\Gamma \right)22}^{-1} \end{array}\right]$$

$$G=\frac{{ZZ}^{\prime }}{2\sum_{i=1}^{m}p_{i}\left(1-p_{i}\right)}$$

where $A_{\left(\Gamma \right)}^{-1}$ and $G^{-1}$ capture the inverse of the pedigree and genomic relationships, respectively. **G** is the genomic relationship matrix which can be calculated using matrix of allele counts **(Z)** and allele frequency (p) of *m* markers. $A_{\left(\Gamma \right)22}^{-1}$ represent the pedigree relationships among genotyped animals which is subtracted from genomic relationships to avoid double-counting of pedigree relationships for genotyped animals. The τ = 1 and ω = 1 are scaling factors used to combine **G** and **A** matrices, respectively (Alvarenga et al., 2020).

### 2.3 Prediction ability (accuracy and dispersion) of purebred parents

The predictive ability of sires in breed A for maximizing CP was evaluated in this study using two metrics, accuracy, and dispersion. Accuracy was assessed according to Esfandyari et al. (2016), by calculating the correlation between the (genomic) estimated breeding value or (G) EBV of sires in breed A and the adjusted CP mean of their crossbred progeny for the number of progenies. The correlation is likely to be higher for sires which have more progeny, as their (G) EBVs are calculated based on performance of large number of progenies. The average accuracy of mean CP was calculated as $\sqrt{\frac{n}{n+k}}$ , where n is the number of progenies per sire and $k=\frac{4-h^{2}}{h^{2}}$ is a correction factor which depends on the heritability of the trait. The predicted dispersion of (G)EBV was measured by the deviation of the slop ( $b_{1}$ ) from the regression TBVs on (G)EBVs in the crossbred population (Kluska et al., 2021).

## 3 Results

### 3.1 Genetic connectedness between populations

**FIGURE 2:**
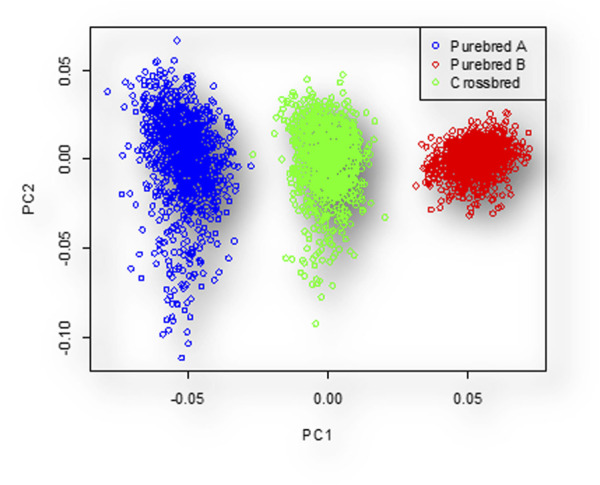
Individuals clustered to three distinct groups based PC1 and PC2. In this figure, the blue, red, and green colors represent the individuals of breed A, breed B, and their crosses, respectively.

The PCA scattered plot is shown in Figure 2. The first two principal components (PCs) explain 8.249% and 7.92% of the total variance, respectively. The genetic relatedness between populations before simulation of $r_{pc}$ levels as measured by $F_{st}$ was 0.032 between purebred parental breeds, while $F_{st}$ between purebred and crossbred populations was 0.023.

### 3.2 Prediction ability

The prediction accuracy of the three models across scenarios is detailed in Table 2. In general, a positive correlation between prediction accuracy and $r_{pc}$ value was observed. However, the BLUP-UPG model exhibited lower accuracy than the other models at all levels of $r_{pc}$ . In contrast, ssGBLUP and ssGBLUP-MF displayed inconsistent trends across their respective scenarios.

**TABLE 2:** Mean and standard deviation (in parentheses) of accuracy across 10 replicates in each scenario with different genetic correlation between crossbred and purebred populations (r_pc_).

$r_{pc}$	Model/Scenario						
	BLUP-UPG	ssGBLUP-MFSC1	ssGBLUP-MFSC2	ssGBLUP-SC1	ssGBLUP-SC2	ssGBLUP-SC3	ssGBLUP-SC4
0.2	0.114 (0.028)	0.204 (0.019)	0.171 (0.028)	0.226 (0.026)	0.195 (0.029)	0.213 (0.022)	0.131 (0.024)
0.5	0.343 (0.023)	0.439 (0.021)	0.424 (0.022)	0.447 (0.022)	0.339 (0.023)	0.405 (0.030)	0.358 (0.018)
0.8	0.505 (0.015)	0.603 (0.014)	0.568 (0.022)	0.618 (0.019)	0.631 (0.019)	0.732 (0.016)	0.545 (0.015)

* indicates the model we used, including model BLUP-UPG, Best Linear Unbiased Prediction with Unknown-Parent Groups; ssGBLUP, Single-Step Genomic BLUP, and ssGBLUP-MF, ssGBLUP, with Metafounders.

** represents the scenarios of genotype selection from purebred and crossbred populations.

Considering the first scenario encompassing genotypes (using breeds A, B, and crossbreds), ssGBLUP outperformed ssGBLUP-MF at all levels of $r_{pc}$ . On the other hand, among the scenarios for selecting genotypes in ssGBLUP, SC3 (using sire genotypes) achieved the highest prediction accuracy. However, the optimal scenario depended on the $r_{pc}$ level. For example, at $r_{pc}$ values of 0.2 and 0.8, ssGBLUP-SC1 and ssGBLUP-SC3 outperformed other scenarios. Interestingly, the scenario incorporating the genotype of purebred dams (SC4) in ssGBLUP had the lowest accuracy among the scenarios, nearly the same as BLUP-UPG. Additionally, at $r_{pc}$ = 0.8, ssGBLUP continued to outperform both ssGBLUP-MF and BLUP-UPG. Specifically, ssGBLUP-SC3 achieved an impressive prediction accuracy of 73.2%.

**FIGURE 3:**
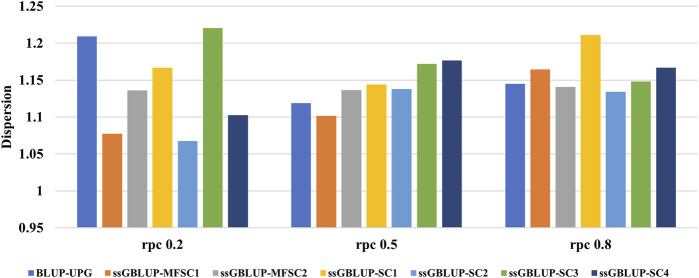
The regression coefficient ( $b_{1}$ ) of TBVs on (G) EBVs for crossbred population across different scenarios and genetic correlation between crossbred and purebred populations (rpc).

In Figure 3, the regression coefficient ( $b_{1}$ ) of TBVs on (G)EBVs in different models is shown which can indicate the dispersion of predicted breeding values. A low regression coefficient ( $b_{1}$ <1.0) indicates overprediction (inflation), and $b_{1}$ >1.0 shows underprediction (deflation) of GEBVs (Tsuruta et al., 2019). The results of our study showed a slight underprediction of)G)EBVs across all scenarios and at different levels of $r_{pc}$ , the regression coefficients ranging from 1.067 to 1.220. A comparison of the regression coefficients between ssGBLUP and ssGBLUP-MF revealed that ssGBLUP-MF predictions had lower dispersion. Furthermore, ssGBLUP-SC2 and ssGBLUP-MFSC1 had minimum dispersion values at the $r_{pc}$ level of 0.2 compared to the other scenarios. At the $r_{pc}$ level of 0.5, ssGBLUP-SC2 exhibited a minor deflation in dispersion, while in contrast, ssBLUP-MFSC1 consistently displayed the lowest dispersion among all the scenarios. At the $r_{pc}$ level of 0.8, the dispersion was almost similar across all scenarios, except ssGBLUP-SC1, which exhibited a slight deflation of the $b_{1}$ compared to the other scenarios.

## 4 Discussions

The genetic merit of a purebred individual can be explained as: (i) its breeding value within the purebred population, and (ii) its breeding value as a purebred parent of a crossbred progeny (Esfandyari et al., 2018). The breeding value of a purebred parent of a crossbred offspring depends on a range of non-additive genetic effects, which can be summarized in $r_{pc}$ . However, it is difficult to consider all factors in $r_{pc}$ simulation. Consequently, in this study, we focused on the overall effect of $r_{pc}$ on evaluating CP based on parental and/or crossbred data.

Based on the PCA graph, we observed three distinct populations. The crossbred population was positioned between the two purebred populations. This positioning was a result of simulating interbreeding within each purebred population for 210 generations, leading to complete differentiation of the two parental lines. The results of the $F_{st}$ analysis also support the findings from the PCA graph. The crossbred individuals genetically possess a combination of traits from both purebred populations, as indicated by the PCA graph. This is because interbreeding was simulated within each purebred population for 210 generations. Extended breeding within each group has resulted in significant genetic differentiation, making the two purebred populations genetically distinct from each other. The $F_{st}$ analysis results, which measure genetic differentiation between populations, align with the PCA graph. High $F_{st}$ values indicate significant differentiation, supporting the observation that the purebred populations are distinct while the crossbred population is intermediate.

The increase in $r_{pc}$ level improved the prediction accuracy in all simulated scenarios. However, the prediction accuracy was not significantly different in different scenarios, which could be due to the method we used to simulate $r_{pc}$ . The BLUP-UPG model showed the lowest prediction accuracy at all $r_{pc}$ levels which was expected, because it completely depended on pedigree relationships. While, genomic evaluation models which uses high-density SNP genotyping can capture relationships between individuals (with or without pedigree connection) more accurately. This may lead to optimal selection of purebred animals in parental lines for improved crossbred performance (Zhao et al., 2015). Our results showed that ssGBLUP-SC1 and ssGBLUP-MFSC1 provide the highest prediction accuracy in all scenarios when the $r_{pc}$ was less than 0.5. Hence, including crossbred and purebred data in the evaluation model could improve CP prediction. However, this improvement was observed only for low $r_{pc}$ value. Which could be useful when there is no emphasis on purebred performance in breeding goal (Van Grevenhof and Van der Werf, 2015), the reference population is large, and the genetic relatedness between crossbred population and purebred selection candidates is high (Wientjes et al., 2020). When the genetic relatedness between purebred and crossbred populations is high, including crossbred data only adds to model complexity and runtime as well as costs of genotyping. The accuracy of CP also depends on the heritability of the trait in purebreds and crossbreds (Wientjes et al., 2020). When the heritability of the trait is lower in crossbreds than purebreds, the accuracy of selection by traditional and genomic evaluations will be adversely impacted.

The ssGBLUP-SC3 model performed the best at the $r_{pc}$ = 0.8, which may be due to the high number of crossbred progenies per sire in breed A. It is important to note that, if purebred QTLs are not included in the combined reference population, accuracy will be significantly reduced. In other words, the SNP effect is primarily determined by breeds that are more common in the reference population when considering the gene flow (Karaman et al., 2021). The linkage disequilibrium between markers and QTLs should also remain high in purebred and crossbred populations. Therefore, using high marker density can be effective in developing prediction equations and preventing loss of accuracy.

Based on the results, ssGBLUP in the first scenario was generally more accurate than ssGBLUP-MF. Although the accuracy of ssGBLUP-MF was expected to be slightly higher than that of ssGBLUP, in our simulated population there were no missing parents, and the base populations of the two purebreds were far apart. In real data, due to considerable number of missing parents in the pedigree, we should not assume the animals with unknown parents are associated with the same base population. Otherwise, the additive variance could not be estimated properly (Mrode, 2014). Therefore, considering pseudo-parents as UPG and metafounders should generally improve the prediction accuracy.

Our results showed a slight deflation of the predicted dispersion of (G)EBV in all scenarios. The predicted dispersion in BLUP-UPG was not significantly different to the other models. This could be due to lack of simulating missing parents and use of UPG in our BLUP-UPG model. Nevertheless, ignoring missing pedigree in BLUP model has been reported to increase the dispersion compared to the genomic models (Bradford et al., 2019). Although, ssGBLUP-MF displayed the lowest dispersion among all the scenarios, incorporation of MF in genomic relationship has only a small impact on the predicted dispersion compared to alternative models. It should be noted that the impact of MF depends on the number of animals with genotypes and phenotypes which are associated with each MF (Kluska et al., 2021). A comparison between MF and UPG revealed that MF predictions had similar or lower levels of inflation and bias (Masuda et al., 2019). Furthermore, ssGBLUP-MF has been reported to be more stable (Kluska et al., 2021). The main difference between UPGs and MFs is that in MFs the relationships within and across populations are take into account to measure the covariance of gametes transmitted from base animals to their descendants (Legarra et al., 2015). However, dispersion in ssGBLUP is noticeable when the pedigree is deep and incomplete, or when inbreeding is ignored in calculation of pedigree relationships (Kluska et al., 2021). Generally, the inflation in breeding values is common when the selected candidates have different amounts of information (Neves et al., 2012). In ssGBLUP, inconsistencies arise from conflicting genomic and pedigree relationship matrices, leading to variations in predictive performance. The inclusion of metafounders (MF) in ssGBLUP-MF aims to address this issue by establishing a unified base population, thereby improving the alignment of genomic and pedigree information. However, due to the simulation process, the performance of the two methods varied across different scenarios, and neither method demonstrated superior performance in all scenarios.

## 5 Conclusions

This study demonstrated that at low $r_{pc}$ levels, employing crossbred and purebred populations in ssGBLUP and ssGBLUP-MF models resulted in enhanced predictive ability for CP. At high $r_{pc}$ levels, utilizing paternal data in the ssGBLUP model yielded optimal CP predictions. The expected slightly higher accuracy of ssGBLUP-MF compared to ssGBLUP was due to the considerable genetic divergence between the base populations of the two simulated purebred populations. Nevertheless, the key factor in maximizing CP based on purebred selection was the $r_{pc}$ value. Although the results can be influenced by $r_{pc}$ and other factors, ultimately, the selection of the most suitable model for enhancing CP depends on $r_{pc}$ . Further research to develop models based on purebred selection to improve CP is warranted.

## Data Availability

The raw data supporting the conclusions of this article will be made available by the authors, without undue reservation.
